# Peripheral exosomal miRNAs as candidate molecular indicators associated with acupuncture at ST36 in an LPS-induced neuroinflammation model

**DOI:** 10.3389/fnmol.2026.1917472

**Published:** 2026-09-15

**Authors:** Seung-Nam Kim

**Affiliations:** Department of Meridian and Acupoint, College of Korean Medicine, Dongguk University, Goyang, Republic of Korea

**Keywords:** acupuncture, biomarker, exosomal microRNA, LPS-induced animal model, neuroinflammation

## Abstract

This study aimed to identify circulating exosomal microRNA (miRNA) candidates showing a control-like expression pattern following ST36 acupuncture treatment in an LPS-induced neuroinflammation model, using empirical Bayes reanalysis of previously published microarray data. Two independent Affymetrix GeneChip miRNA 4.0 microarray datasets from C57BL/6 male mice were reanalysed using the limma empirical Bayes framework with Benjamini-Hochberg false discovery rate (FDR) correction. After detection-call filtering, 286 commonly expressed probes were identified. Control-like pattern candidates were defined by three criteria: FDR < 0.05 for the LPS effect, FDR > 0.05 for the LPS+ST36 vs CON comparison, and logFC direction opposite to the LPS effect. Target gene prediction and pathway enrichment were subsequently performed. Eight miRNA candidates were identified: five upregulated by LPS and showing a control-like pattern following ST36 (let-7d-5p, miR-103-3p, miR-107-3p, miR-451a, miR-7047-5p) and three downregulated by LPS and showing a control-like pattern following ST36 (miR-5128, miR-16-2-3p, miR-101a-5p). None overlapped with candidates from the original fold-change-based analysis. Functional enrichment of 1,596 predicted target genes revealed significant enrichment in MAPK, PI3K-Akt, and Wnt signaling pathways, with Gene Ontology analysis highlighting convergence on neuronal and synaptic processes. Limma-based reanalysis reveals a distinct set of ST36-associated exosomal miRNAs overlooked by threshold-based approaches, providing exploratory candidate molecular indicators for further investigation of acupuncture's peripheral effects in neuroinflammation.

## 1 Introduction

Identifying the molecular mechanisms of acupuncture has largely relied on brain tissue analyses, which are inherently invasive and difficult to translate into clinical monitoring. Circulating exosomal miRNAs offer a compelling alternative: stable in peripheral blood, reflective of systemic inflammatory states, and accessible without tissue sampling (Nik Mohamed Kamal and Shahidan, 2019). Their role as peripheral sentinels of neuroinflammatory processes in central nervous system diseases has gained increasing attention (Xia et al., 2019), yet their connection to acupuncture's therapeutic effects remains poorly characterized. MicroRNAs (miRNAs) are small non-coding RNAs that broadly regulate post-transcriptional gene expression and have emerged as key modulators of inflammation and innate immune responses (Nejad et al., 2018; Gaudet et al., 2018). Exosomal packaging confers additional stability in circulation, making them attractive candidates as peripheral biomarkers of disease state and treatment response (Nik Mohamed Kamal and Shahidan, 2019). Acupuncture at ST36 (Zusanli) is well-established as a modulator of neuro-immune pathways, including the vagal-adrenal anti-inflammatory axis (Liu et al., 2021; Torres-Rosas et al., 2014), with documented efficacy in modulating systemic inflammation across multiple conditions (Li et al., 2021; Pan et al., 2021). The association between acupuncture stimulation and miRNA changes has been reported in various disease models (Ko and Kim, 2019; Deng et al., 2017), yet the specific peripheral miRNA signatures underlying ST36's therapeutic effects in neuroinflammation remain incompletely defined. Furthermore, it has been argued that the anti-inflammatory mechanism of electroacupuncture involves modulation of multiple systems and targets beyond the vagal-adrenal axis alone (Fan, 2023), further underscoring the need for comprehensive molecular characterization.

We recently reported alterations in serum exosomal miRNAs in an LPS-induced depression model treated with ST36 acupuncture, providing preliminary evidence that peripheral miRNA profiles may reflect acupuncture-induced immunomodulation (Ko et al., 2025). However, candidate identification in that study relied on fold-change thresholds and unpaired t-tests without formal statistical validation across independent datasets, limiting the robustness of those findings. The empirical Bayes framework implemented in the limma package offers substantially improved statistical power for small-sample microarray studies by moderating per-probe variance estimates toward a common prior — an advantage particularly relevant given the *n* = 3 per group design of the original data (Ritchie et al., 2015).

Here, we apply limma-based reanalysis to the same datasets to identify exosomal miRNA candidates showing a control-like expression pattern following ST36 treatment, and characterize their predicted functional targets. This represents a step toward identifying exploratory candidate molecular indicators of acupuncture response in neuroinflammatory conditions.

## 2 Methods

### 2.1 Data sources

Two independent Affymetrix GeneChip miRNA 4.0 microarray datasets derived from C57BL/6 male mice were reanalysed, as previously described (Ko et al., 2025). The first dataset comprised individual serum exosome samples from three groups: CON (control), ST36 (ST36 acupuncture only), and LPS+ST36 (LPS-induced inflammation with ST36 treatment), with *n* = 3 per group. The second dataset comprised CON and LPS groups (*n* = 3 per group). Although both datasets were generated in the same laboratory using the same array platform, reagent lot, and mouse strain, they were processed as separate microarray batches; cross-dataset comparability was therefore assumed rather than formally verified, and residual batch effects cannot be entirely excluded. All animal procedures in the original study from which the archived microarray data were generated were conducted in accordance with institutional guidelines approved by the Dongguk University Animal Care Committee (DGU-IACUC-2021-055-1). No new animal experiments were performed for the present reanalysis.

### 2.2 Data preprocessing and filtering

Individual sample log2-transformed expression values were extracted from each dataset following raw data extraction using the Affymetrix data extraction protocol in the AGCC software, after which background subtraction, normalization, and probe-set summarization were performed using the miRNA-level RMA+DABG algorithm in Affymetrix^®^ Power Tools (APT), as previously described (Ko et al., 2025). Array data were filtered using probe-annotated species (*Mus musculus*). Detection status was defined using the DABG (Detected Above Background) p-value; probes were retained for the present reanalysis if DABG *p* < 0.05 was observed in at least 2 of 3 samples within at least one experimental group, yielding 423 expressed probes in the first dataset and 352 in the second, with 286 common probes used for cross-dataset analysis. Probe annotation was based on the manufacturer-provided Affymetrix miRNA 4.0 array annotation (miRBase release 22); each probe set corresponded to a single unique mature miRNA, with no instances of multiple probe sets mapping to the same mature miRNA (Supplementary Table S1).

### 2.3 Differential expression analysis

Differential expression was assessed using the limma empirical Bayes framework (Ritchie et al., 2015) with Benjamini-Hochberg FDR correction (Benjamini and Hochberg, 1995), implemented in R (v4.5.1). For each dataset, a design matrix without intercept (−0 + group) was fitted using lmFit (), followed by contrasts.fit () with the contrasts specified below and eBayes () with robust = TRUE and trend = FALSE (robust empirical Bayes shrinkage was used to moderate the influence of outlier probes on the variance prior). For the second dataset, the contrast of interest was LPS vs CON. For the first dataset, three contrasts were examined: ST36 vs. CON, LPS+ST36 vs. CON, and LPS+ST36 vs ST36 (Supplementary Table S2 and S6). The full design and contrast matrix specification, along with the analysis code, are provided in the deposited repository.

### 2.4 Normalization candidate definition

Control-like pattern candidates were defined among the 286 common probes as those satisfying all three criteria simultaneously: (1) FDR < 0.05 in the LPS vs. CON comparison (LPS effect confirmed), (2) FDR > 0.05 in the LPS+ST36 vs. CON comparison (expression not statistically distinguishable from control), and (3) logFC direction in LPS+ST36 vs. CON opposite to the LPS effect direction. This three-criterion definition was intentionally conservative, selecting only miRNAs for which the LPS-induced change was no longer statistically distinguishable from control levels after ST36 treatment; miRNAs that were only partially normalized (i.e., significantly altered in both the LPS-vs-CON and LPS+ST36-vs-CON comparisons but with an attenuated effect size) were not captured by this definition and may nonetheless be biologically and therapeutically relevant. Because criterion (2) reflects failure to reject the null hypothesis rather than a formal test of equivalence, these candidates are described throughout as showing a “control-like expression pattern” rather than as statistically demonstrated normalization.

### 2.5 Target gene prediction and pathway enrichment

Predicted target genes for the eight candidate miRNAs were identified using miRNAtap (v1.46.0, miRNAtap.db v0.99.10) with the geometric-mean rank aggregation method and a minimum two-source consensus requirement (species: mmu), integrating predictions from miRDB (Chen and Wang, 2020), TargetScan, PicTar, DIANA-microT, and miRanda. Target genes were pooled across all eight miRNAs prior to enrichment analysis (1,596 unique Entrez-annotated genes); this pooled approach was chosen for the exploratory functional characterization presented here but is noted as a limitation in the Discussion, since it precludes attribution of specific pathways to individual miRNAs. Pathway enrichment was performed using clusterProfiler (v4.20.0) (Wu et al., 2021) with the KEGG mmu pathway database (enrichKEGG) and the Gene Ontology Biological Process database via org.Mm.eg.db (v3.23.0) (enrichGO), using the default clusterProfiler background gene universe (all annotated genes in org.Mm.eg.db) and Benjamini-Hochberg-adjusted *p* < 0.05 as the significance threshold. Analyses were performed in R (v4.6.0); the eight target-prediction and enrichment results are provided in full in Supplementary Tables S3 (KEGG, all 57 significant pathways) and S4 (GO Biological Process, all 1,193 significant terms), with GO term redundancy reduction (simplify(), similarity cutoff 0.7) applied for the reduced term set shown in Figure 1D. As a sensitivity analysis, target prediction was repeated using only experimentally validated miRNA-target interactions retrieved via the multiMiR package (v1.34.0, database accessed August 20, 2026, validated tables including miRTarBase, miRecords, and TarBase), yielding 8,357 unique validated target genes across the eight candidates. KEGG and GO enrichment were repeated on this validated-target set using identical parameters; 55 of the 57 KEGG pathways identified using predicted targets were also significantly enriched using validated targets alone, indicating that the predicted-target-based enrichment results are largely robust to restriction to experimentally confirmed interactions. Full validated-target enrichment results are provided in Supplementary Table S7.

**Figure 1:**
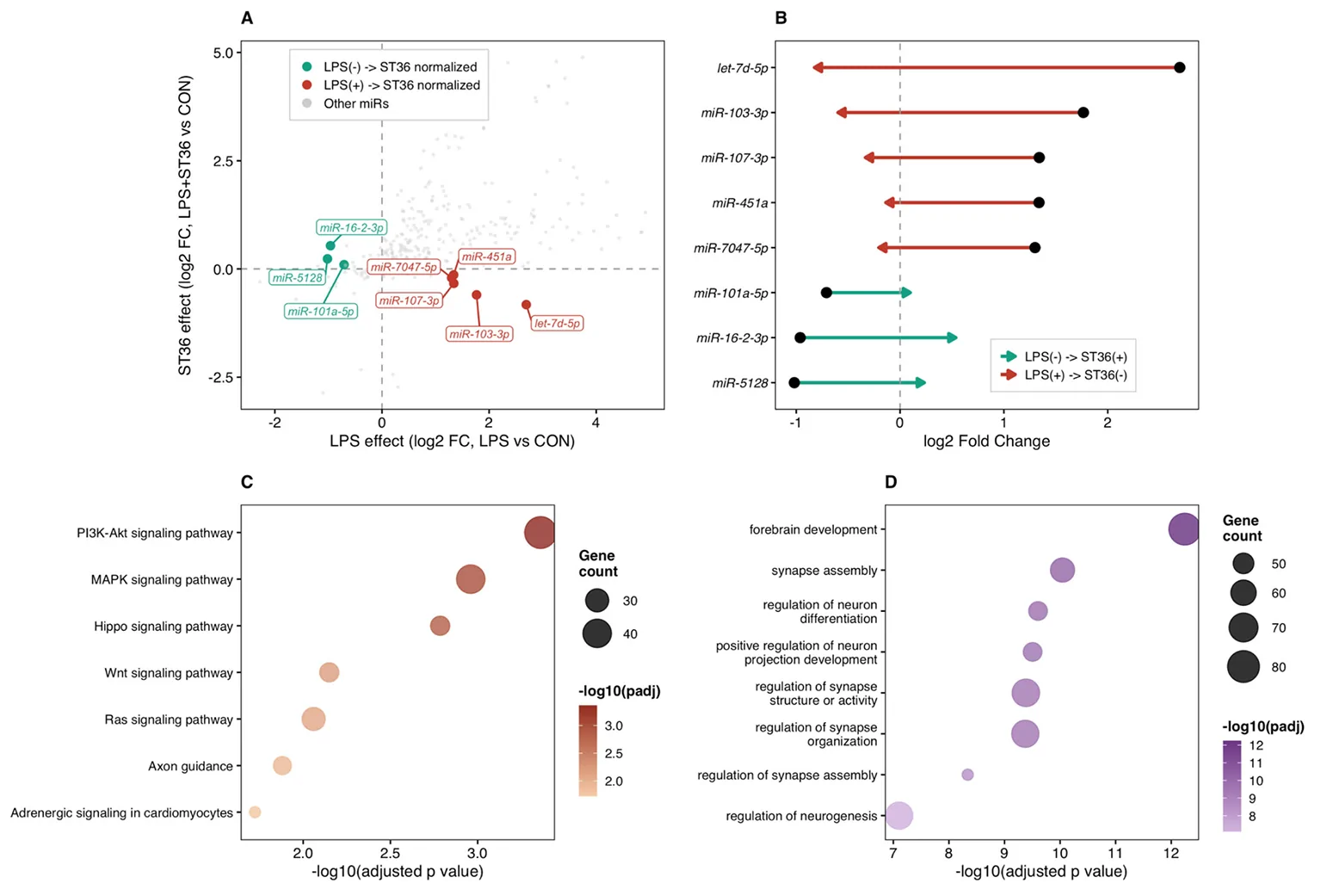
Identification and functional characterization of exosomal miRNA candidates showing a control-like expression pattern following ST36 treatment. **(A)** Scatter plot of LPS-induced (x-axis) versus ST36 treatment effects (y-axis) across 286 commonly expressed miRNA probes. Eight control-like pattern candidates are highlighted: miRNAs upregulated by LPS and showing a control-like pattern following ST36 (red) and miRNAs downregulated by LPS and showing a control-like pattern following ST36 (teal). **(B)** Arrow plot illustrating the direction and magnitude of expression change for each candidate. Filled circles represent the LPS effect (LPS vs CON, second dataset); arrowheads indicate the ST36 treatment effect (LPS+ST36 vs CON, first dataset). As these two effects were estimated from independent datasets (see Methods 2.1), the arrows depict a cross-dataset comparison of effect sizes and should not be interpreted as a within-experiment reversal trajectory. **(C)** Bubble plot of the top significantly enriched KEGG pathways among predicted target genes of the eight candidates (BH-adjusted p < 0.05), selected by adjusted p-value for display; the complete list of all 57 significant pathways is provided in Supplementary Table S3. **(D)** Bubble plot of the top Gene Ontology Biological Process terms after redundancy reduction (semantic similarity cutoff 0.7), highlighting convergence on neuronal and synaptic processes; the complete list of all 1,193 significant terms is provided in Supplementary Table S4. Bubble size indicates gene count; color intensity reflects statistical significance. A color-blind-accessible palette (Okabe-Ito) is used throughout.

## 3 Results

### 3.1 Differential expression analysis

Limma reanalysis of the LPS vs. CON comparison identified 123 FDR-significant probes among 352 expressed probes. In the first dataset, the LPS+ST36 vs. CON comparison yielded 49 FDR-significant probes among 423 expressed, while ST36 vs. CON yielded 0 FDR-significant probes. Given the limited power of this *n* = 3-per-group comparison, this null result should not be interpreted as evidence that ST36 has no effect on miRNA expression in healthy controls (absence of evidence is not evidence of absence); point estimates and 95% confidence intervals for this comparison are provided in Supplementary Table S5.

### 3.2 Control-like pattern candidates

Applying the three-criterion control-like pattern definition to the 286 common probes yielded eight candidates (Figure 1A, B). Five were upregulated by LPS and showed a control-like pattern following ST36 treatment: mmu-let-7d-5p (LPS logFC = + 2.69), mmu-miR-103-3p (+ 1.77), mmu-miR-107-3p (+ 1.34), mmu-miR-451a (+ 1.34), and mmu-miR-7047-5p (+ 1.30). Three were downregulated by LPS and showed a control-like pattern following ST36 treatment: mmu-miR-5128 (LPS logFC = −1.02), mmu-miR-16-2-3p (−0.96), and mmu-miR-101a-5p (−0.71). None of these eight candidates overlapped with the miRNAs identified in the original fold-change-based analysis (Ko et al., 2025) (Supplementary Table S8), indicating that statistical methodology can substantially influence candidate selection in small-sample miRNA studies.

### 3.3 Functional enrichment

Target gene prediction yielded 1,596 unique predicted targets (full results in Supplementary Table S3 and S4). KEGG pathway enrichment identified 57 significant pathways, with the strongest enrichment in PI3K-Akt signaling (p.adj = 0.0004), MAPK signaling (p.adj = 0.0011), Hippo signaling (p.adj = 0.0016), and Wnt signaling (p.adj = 0.0071; Figure 1C). Axon guidance was also significantly enriched (p.adj = 0.0132); together with the Gene Ontology results below, this pattern is compatible with, though does not by itself establish, a neuroplasticity-related mechanism. Gene Ontology analysis revealed 1,193 significant Biological Process terms, with the strongest enrichment in forebrain development, synapse assembly, regulation of neuron differentiation, and regulation of synapse structure or activity (all p.adj < 10^−9^; Figure 1D). As a sensitivity analysis restricted to experimentally validated miRNA-target interactions (8,357 unique validated targets), 55 of the 57 predicted-target-based KEGG pathways remained significantly enriched, indicating that these enrichment results are largely robust to restriction to validated interactions (Supplementary Table S7).

## 4 Discussion

The eight control-like pattern candidates identified here were entirely invisible to the original fold-change threshold approach, underscoring the practical importance of statistical methodology in small-sample miRNA studies. The limma empirical Bayes framework improves sensitivity by moderating per-probe variance estimates toward a common prior (Ritchie et al., 2015), with robust empirical Bayes shrinkage further limiting the influence of outlier probes on this prior, a critical advantage when small sample size per group renders individual variance estimates unreliable.

Among the candidates, let-7d-5p and miR-103-3p are well-established regulators of neuroinflammatory signaling (Gaudet et al., 2018). The control-like pattern observed for these miRNAs following ST36 treatment is consistent with acupuncture's documented modulation of neuro-immune pathways, including via the vagal-adrenal axis described for electroacupuncture (Liu et al., 2021) and broader immunomodulatory effects reported across acupuncture modalities (Li et al., 2021; Pan et al., 2021); as the present study used manual acupuncture, this comparison is drawn by analogy rather than as direct mechanistic evidence (see also Methods 2.1). The enrichment of MAPK and PI3K-Akt signaling pathways among predicted targets—robust to restriction to experimentally validated interactions (Results 3.3)—is compatible with a candidate, bioinformatically-inferred pathway linking ST36 stimulation to neuroplasticity-related gene networks in the context of LPS-induced neuroinflammation, though this remains hypothesis-generating and has not been experimentally confirmed. The convergence of Gene Ontology terms on synaptic and neuronal processes is similarly consistent with, though does not independently establish, a neuroinflammatory mechanism relevant to the depression-related phenotypes reported in the source model (Afridi and Suk, 2021).

A notable observation is that ST36 produced no FDR-significant miRNA changes in healthy controls, in contrast to the 49 FDR-significant probes observed in the LPS+ST36 vs CON comparison. Given the limited power of the n=3-per-group ST36 vs CON comparison (Results 3.1), this contrast should be interpreted cautiously and does not, by itself, establish that the candidate miRNAs are specific to an inflammatory context; it is compatible with, but does not confirm, context-dependent biological activity rather than a non-specific needle effect. Confirming this pattern will require adequately powered comparisons and, ultimately, functional validation, before any claim of biological specificity relevant to biomarker candidacy can be made.

The translational significance of focusing on circulating exosomal miRNAs, rather than brain tissue, lies in their accessibility (Nik Mohamed Kamal and Shahidan, 2019; Xia et al., 2019). Peripheral blood sampling is feasible in clinical settings, and exosomal miRNA stability makes them tractable as monitoring tools (Nik Mohamed Kamal and Shahidan, 2019). If these candidates generalize to other inflammatory models or to human acupuncture studies, they may provide candidate molecular endpoints for future validation in acupuncture treatment-response studies. Such generalization will also require attention to interspecies conservation: several candidates identified here, including mmu-miR-7047-5p and mmu-miR-5128, are mouse-specific or have limited characterization of human orthologs, whereas let-7d-5p and miR-103-3p are broadly conserved across mammals, and this distinction should be systematically evaluated before translational application.

Important limitations must be acknowledged. The small sample size limits statistical power and precludes characterization of individual variability. The analysis is correlational and exploratory; no *in vitro* or *in vivo* functional validation of the identified candidates or their predicted targets was performed. The absence of a LPS-only group in the first dataset required cross-dataset integration, which assumes comparable experimental conditions across batches processed separately (see Methods 2.1); residual batch effects cannot be excluded. This study used only male mice, such that findings may not generalize to females. No independent replication dataset was available, and the candidate miRNAs have not been technically validated by an orthogonal method such as RT-qPCR. These candidates require confirmation in larger, independent, and adequately powered cohorts, together with experimental and technical validation, before mechanistic conclusions can be drawn. Nevertheless, the identification of circulating exosomal miRNA candidates through statistical reanalysis offers a non-invasive window into acupuncture's therapeutic mechanisms, an approach with translational advantages over brain tissue-based analyses, and a potential basis for future development of peripheral molecular indicators of treatment response in neuroinflammatory conditions.

## 5 Conclusions

Limma-based reanalysis of exosomal miRNA microarray data identifies eight ST36-associated control-like pattern candidates not detected by the original fold-change approach. Their predicted targets converge on neuroinflammatory and neuroplasticity-related pathways, a pattern compatible with, though not proof of, a mechanistic link to ST36 acupuncture in the LPS-induced neuroinflammation model used here. These findings highlight the importance of statistical methodology in small-sample miRNA studies and identify exploratory candidate molecular indicators warranting further validation for peripheral biomarker development in acupuncture research.

## Data Availability

All data and analysis materials supporting the findings of this study, including datasets, analysis code, results, figures, and tables, are publicly available in the Zenodo repository at https://doi.org/10.5281/zenodo.22031006.
